# Hemodialysis patients perceived exercise benefits and barriers: the association with health-related quality of life

**DOI:** 10.1186/s12882-021-02292-3

**Published:** 2021-03-16

**Authors:** Mansour Ghafourifard, Banafshe Mehrizade, Hadi Hassankhani, Mohammad Heidari

**Affiliations:** 1 Department of Medical Surgical Nursing, Faculty of Nursing and Midwifery, Tabriz University of Medical Sciences, Shariati-jonubi St., Tabriz, 4515789589 Iran; 2 Community-Oriented Nursing Midwifery Research Center, Shahrekord University of Medical Sciences, Shahrekord, Iran

**Keywords:** Hemodialysis patients, Exercise benefits and barriers, Health-related quality of life

## Abstract

**Background:**

Patients on hemodialysis have less exercise capacity and lower health-related quality of life than healthy individuals without chronic kidney disease (CKD). One of the factors that may influence exercise behavior among these patients is their perception of exercise benefits and barriers. The present study aimed to assess the perception of hemodialysis patients about exercise benefits and barriers and its association with patients’ health-related quality of life.

**Methods:**

In this cross-sectional study, 227 patients undergoing hemodialysis were randomly selected from two dialysis centers. Data collection was carried out using dialysis patient-perceived exercise benefits and barriers scale (DPEBBS) and kidney disease quality of life short form (KDQOL-SF). Data were analyzed using SPSS software ver. 21.

**Results:**

The mean score of DPEBBS was 68.2 ± 7.4 (range: 24 to 96) and the mean KDQOL score was 48.9 ± 23.3 (range: 0 to 100). Data analysis by Pearson correlation coefficient showed a positive and significant relationship between the mean scores of DPEBBS and the total score of KDQOL (*r* = 0.55, *p* < 0.001). Moreover, there was a positive relationship between the mean scores of DPEBBS and the mean score of all domains of KDQOL.

**Conclusion:**

Although most of the patients undergoing hemodialysis had a positive perception of the exercise, the majority of them do not engage in exercise; it could be contributed to the barriers of exercise such as tiredness, muscle fatigue, and fear of arteriovenous fistula injury. Providing exercise facilities, encouraging the patients by the health care provider to engage in exercise programs, and incorporation of exercise professionals into hemodialysis centers could help the patients to engage in regular exercise.

## Introduction

Chronic diseases are the main challenges in health systems that impose enormous healthcare costs for societies and governments [1, 2]. Chronic kidney disease (CKD), as a chronic disease, is considered a major health problem worldwide [3]. An irreversible decrease in kidney functions among patients with CKD ultimately progresses to end-stage kidney disease (ESKD) [4, 5].

There are about 3,730,000 patients with CKD worldwide and the annual growth of this disease is 5–6% [6]. According to the statistics, there are about 2.5 million ESKD patients who receive renal replacement therapy (RRT), and this population is expected to double to about 4.5 million by 2030 [7]. Hemodialysis is the most common method of RRT for patients suffering from CKD [8]. In Iran, about 30,800 patients are undergoing hemodialysis [6].

Although the rapid development of hemodialysis technology could lead to a significant increase in the life expectancy of patients with ESKD and this modality alleviates uremic symptoms of CKD [5, 9], it does not change the process of the underlying disease. Thus, the patients suffer from some complications such as anemia, decreased aerobic capacity, imbalance in body homeostasis [10], decreased muscle strength and function [11], and some infections and malignant neoplasms [12]; which all of them could lead to reduced physical activity [13], increase in duration and number of hospitalizations [14], and impose high costs on patients and health care systems [15]. Ultimately, all these problems and complications could have a negative impact on the health-related quality of life (HRQoL) of patients undergoing hemodialysis [16].

One of the effective strategies to control or eliminate some of the dialysis complications is the use of exercise [17]. There is a difference between physical activity and exercise. Physical activity refers to any body movements produced by the contraction of skeletal muscles. But, exercise is a physical activity that is structured, planned, repetitive, and purposeful [18]. The exercise not only reduces the complications of hemodialysis [19] but also decreases the mortality rate of these patients [20]. Exercise can be defined as any physical activity, including walking, mountain climbing, stair climbing, etc. performed by hemodialysis patients that can improve physical fitness and aerobic capacity [21]. Exercise is beneficial to the physical health of dialysis patients [22] and improves cardiovascular function, blood pressure, muscle strength, nutritional status, and dialysis quality. It also reduces negative emotions such as anxiety and depression, makes them feel better, and improves the social interaction of patients and their families [23, 24]. Therefore, it is necessary to evaluate the physical activity of patients undergoing hemodialysis and to encourage them to engage in an exercise in their life [25].

Although the benefits of exercise for patients on hemodialysis are well documented, many patients do not engage in regular exercise. Moreover, these patients have less physical ability and exercise capacity than healthy people without CKD [26, 27]. Segura-Ortí et al. [28] compared the physical functioning among three groups including patients with no CKD, Stages 3 to 4 CKD, and hemodialysis. The results showed that physical activity in patients with stage 3 or 4 CKD was lower than controls and was similar to hemodialysis patients. The evidence shows that only 6% of hemodialysis patients have physical activity of 4 to 5 days a week [20]. Segura-Ortí et al. [28] argued that nephrology nurses should promote interventions at the clinical setting aimed to improve the physical activity of patients with CKD.

One of the factors that may affect exercise activities among dialysis patients is their perception of the exercise benefits and barriers [29]. Perceived exercise benefits are defined as various beliefs regarding positive outcomes of exercise, and perceived exercise barriers refer to patients’ negative beliefs that prevent them from engaging in exercise and physical activities. It seems that a greater perceived benefit from exercise could lead to greater participation in physical activities, while greater perceived barriers from exercise may lead them to avoid exercise participation [24, 29]. According to the literature review, some of the perceived benefits of exercise include better control of diabetes and blood pressure, improved heart rate changes, nutrition and mental health [25], improved physical function, physical capacity and physical fitness [20], prevention of falls [22], and improved sleep quality [21]. In contrast, the factors such as the presence of ESKD symptoms, exercise-related adverse outcomes [13], underlying diseases, psychological factors [27], socio-economic and cultural factors such as low literacy, low income, lack of access to exercise facilities, lack of motivation and interest [30], old age, exacerbation of dialysis-related symptoms [26], absence of support for exercise from family, friends, and health care providers [22], and insufficient patients’ knowledge of exercise benefits [31]. Moreover, fatigue has been reported as the main barrier to exercise in patients undergoing hemodialysis [31, 32].

One of the important variables in the care of patients undergoing hemodialysis is the health-related quality of life of these patients. The evidence shows that health-related quality of life and daily physical activity of patients undergoing hemodialysis is unsatisfactory as compared to normal individuals, and most of them experience complications such as decreased physical function, anxiety, and depression [33, 34]. According to the definition of world health organization (WHO), health-related quality of life is a state of complete physical, mental, and psychosocial well-being and does not merely the absence of disease or disability. Health-related quality of life is a person’s perception of his/her situation, which is determined by cultural factors, goals, and beliefs of the individual [35]. It could be influenced by demographics, social variables, and diseases related factors [35, 36]. According to the literature review, the sedentary lifestyle is more common among dialysis patients and they have less physical ability and exercise capacity than healthy people [31, 37]. Considering the relationship between inactivity and increased mortality among dialysis patients, it is not known why CKD patients don’t do the exercises [30]. One of the influential factors, which may contribute to the physical activity and healthy lifestyle of hemodialysis patients, is patients’ perception of exercise benefits and barriers. These factors are context-based and may differ based on patients’ conditions, socio-cultural factors, and access to exercise facilities. Therefore, there is a need to carry out further studies in different cultures. Moreover, the relationship between patients’ perception of exercise benefits and barriers and their QoL has not been studied yet. Therefore, this study aimed to assess the hemodialysis patients’ perception of exercise benefits and barriers and its association with health-related quality of life.

## Methods

### Design

This is a cross-sectional study that was performed on 227 patients undergoing hemodialysis in two hemodialysis units of educational hospitals (Imam Reza and Sina Hospitals) affiliated to Tabriz University of Medical Sciences, Iran. These two hemodialysis units admit the majority of patients on hemodialysis in the northwest of Iran. All methods were performed in line with the STROBE Statement as a relevant guideline for cross-sectional studies.

### Sample and setting

Patients were selected using a random sampling method. Inclusion criteria included age over 18 years, no mobility restrictions for exercise according to physician order, being treated with maintenance hemodialysis for at least 6 months, receiving hemodialysis at least twice a week, and willingness to participate in the study. The informed consent was obtained from all participants or from a parent and/or the legal guardian.

### Data collection

Data were collected by two following tools;

1- Dialysis Patient-Perceived Exercise Benefits and Barriers Scale (DPEBBS): It was developed by Zheng et al. [23] and consists of 24 questions, 12 of which focus on exercise benefits and the remaining 12 questions on exercise barriers. The answers to questions on exercise benefits were scored based on a 4-point Likert scale including (1 = Strongly disagree, 2 = Disagree, 3 = Agree, and 4 = Strongly agree) while answers to questions on exercise barriers were scored reversely (1 = Strongly agree, 2 = Agree, 3 = Disagree and 4 = Strongly disagree). Therefore, the total score ranged from 24 to 96. The higher scores indicate a greater perception of exercise benefits and lower perception of exercise barriers; and conversely, lower scores indicate a lower perception of exercise benefits and higher perception of exercise barriers. Moreover, two open-ended questions asked patients to describe other benefits and barriers to exercise not included in the scale.

The validity and reliability of this instrument have been investigated in previous studies using Cronbach’s alpha (α = 0.84) [23]. In this study, the validity of the Persian version of the scale was assessed by content validity. For this purpose, after being translated from English to Persian and back-translated to English by a bilingual expert, the questionnaire was provided to 10 faculties of the nursing to check the content validity of the scale. After receiving their comments, the necessary changes were made on the scale. Moreover, the reliability of the instrument was assured by Cronbach’s alpha coefficient of 0.87.

2- Kidney disease quality of life short form (KDQOL-SF™) was used to measure QoL of CKD patients on hemodialysis. This questionnaire was developed by Hays et al. [38] in 1994. The instrument consisted of 36 questions consisting of 4 domains including symptoms/problems (12 items), effects of kidney disease on daily life (8 items), the burden of kidney disease (4 items), and SF-12 (12 items). Multiple-choice questions are used in this scale and the patient must choose one of the options. The final score shows the QoL of CKD patients. The possible range of scores in each domain is 0 to 100, and a score above 50 in any domain indicates a better health-related quality of life.

Hays et al. assessed the reliability of the scale and reported the Cronbach’s alpha of above 0.70 for each domain [38]. The psychometric properties of the Persian version of KDQOL-SF™ have been assessed by Yekaninejad et al. [39] in Iran with a Cronbach’s alpha ranged between 0.73 to 0.93, which indicates the high reliability of the instrument [39]. In our study, Cronbach’s alpha coefficient for this instrument was 0.93.

In addition to the questionnaires, patient demographics and medical information such as duration of hemodialysis, the number of hemodialysis sessions per week, etc. were also recorded.

### Data analysis

Data analysis was carried out by SPSS (version 21.0, SPSS Inc., Chicago, IL) software. Descriptive statistics including mean, standard deviation, and ranges (means and medians) were used to describe participants’ demographics or variables. Moreover, the Pearson correlation test was used to assess the correlation between patients’ perceived exercise benefits and barriers and patients’ Qol. The Kolmogorov-Smirnov test was used to check the normality distribution before applying Pearson correlation. *P*-value< 0.05 was considered a statistical significance level.

## Results

### Descriptive characteristics

A total of 227 hemodialysis patients participated in this study. The results showed that the mean age of participants was 57.9 ± 15.3 years and the majority of them (63.9%) were male. About 77.5% of the participants were married and the majority of them (96%) lived in urban. In term of educational levels, 33.9% of them were illiterate. Approximately, half of the participants (47.1%) had a monthly income of fewer than 10 million Rials. Most of the patients (72.2%) were undergoing hemodialysis three times a week (Table 1).

**Table 1:** Demographic and clinical characteristics of patients (*N* = 227)

Variables			Mean	SD
			N	%
Age (years)			57.9	15.3
Gender	Male		145	63.9
	Female		82	36.1
Marital status	Single		19	8.4
	Married		176	77.5
	Divorced		2	0.9
	Widow		30	13.2
Education level	Illiterate		77	33.9
	Elementary school		57	25.1
	Junior High school		24	10.6
	Senior Diploma		35	15.4
	University		34	15
Employment status	Employee		5	2.2
	Teacher		1	0.4
	Retired		46	20.3
	military		2	0.9
	Worker		4	1.8
	Self- employment		49	21.6
	Homemaker		71	31.3
	Unemployed		49	21.5
	Mean ± sd			
Duration of hemodialysis treatment (years)	3.3 ± 3.0			
	Yes		No	
	N	%	N	%
Are you able to perform the daily activities independently?	170	74.9	57	25.1
Do you engage in physical activities?	82	36.1	145	63.9
Do you have exercises equipment in your home?	10	4.4	217	95.6
Are there exercise facilities near your home?	65	28.6	162	71.4

According to the findings, 74.9% of the samples were able to perform their daily activities independently, but the majority of them (63.9%) do not engage in any physical activities. A total of 95.6% of the patients told that they do not have exercise equipment in their home and 71.4% of the participants stated that there are no exercise facilities near their home. As shown in Table 2, the most common cause of CKD in patients was hypertension (30%) and diabetes mellitus (26.8%).

**Table 2:** Etiology of end stage kidney disease in patients undergoing hemodialysis

Etiology	N	%
Hypertension	68	30
Diabetes	61	26.8
Diabetes and Hypertension	22	9.6
Drug side effects	12	5.3
Autoimmune disease	5	2.2
Inherited	2	0.9
Polycystic kidney disease	14	6.2
Glomerulonephritis	24	10.6
Others	19	8.4

### Patients’ perception on benefits and barriers of exercise

Table 3 shows the mean score of DPEBBS. According to the result, the mean score of DPEBBS was 68.2 ± 7.4 (in a possible range of 24 to 96). The participants perceived the factors such as “exercise improves my mood (3.5± 0.6)”, “exercise prevents muscular wasting (3.3±0.6)”, and “exercise improves my appetite (3.2±0.5)” as the main benefits of exercise. In contrast, frequent tiredness (2.3 ± 0.7), frequent lower-extremity muscle fatigue (2.3 ± 0.6), and fear of arteriovenous fistula injury (2.3 ± 0.6) were perceived as main barriers to exercise participation (Table 3). In response to two open-ended questions of DPEBBS, some participants referred to other barriers to exercise such as lack of exercise facilities at home or near their home, and lack of encouragement by hemodialysis staff to exercise.

**Table 3:** Dialysis patient-perceived Exercise Benefits and Barriers Scale (DPEBBS)

Items	Range	Mean	SD
Q1- Exercise helps reduce my total medical costs.	**1–4**	**2.8**	**0.7**
Q2- Exercise helps reduce my body pain.	**1–4**	**3.2**	**0.6**
Q3- Exercise can postpone a decline in body function.	**1–4**	**3.2**	**0.7**
Q4- Exercise prevents muscular wasting.	**1–4**	**3.3**	**0.6**
Q5- Frequent tiredness impedes my exercise participation.	**1–4**	**2.3**	**0.7**
Q6- Exercise improves my mood.	**1–4**	**3.5**	**0.6**
Q7- Exercise improves bone disease.	**1–4**	**2.9**	**0.7**
Q8- Exercise is adverse to health of dialysis patients.	**1–4**	**3.0**	**0.4**
Q9- I worry about a fall during exercise.	**1–4**	**2.5**	**0.7**
Q10 Exercise improves my appetite.	**1–4**	**3.2**	**0.5**
Q11- Frequent lower-extremity muscle fatigue impedes my exercise participation.	**1–4**	**2.3**	**0.6**
Q12- I lack an understanding of the benefits of exercise.	**1–4**	**2.9**	**0.6**
Q13- Exercise helps me to lead an optimistic and active life.	**1–4**	**3.2**	**0.5**
Q14- Exercise is not suitable for me since I have other medical conditions.	**1–4**	**2.5**	**0.7**
Q15- Body pain impedes my exercise participation.	**1–4**	**2.4**	**0.7**
Q16- Exercise improves my quality of life.	**1–4**	**3.1**	**0.5**
Q17- I lack an understanding of the knowledge on how to carry out exercise.	**1–4**	**2.5**	**0.7**
Q18- I worry that exercise may make me feel thirsty.	**1–4**	**2.6**	**0.7**
Q19- Exercise is not suitable for me since I have kidney disease.	**1–4**	**2.7**	**0.6**
Q20- Exercise can keep my body weight at a steady level.	**1–4**	**3.0**	**0.5**
Q21- I worry that exercise may affect my arteriovenous fistula	**1–4**	**2.3**	**0.6**
Q22- Exercise helps enhance my self-care abilities.	**1–4**	**3.1**	**0.4**
Q23- Exercise will keep me free from having other diseases (e.g., cold).	**1–4**	**2.3**	**0.5**
Q24- Outdoor exercise adds burden to my family (since I need their company while I am out).	**1–4**	**2.5**	**0.7**
		Mean	SD
Total score of DPEBBS (in a range 44–90)		68.2	7.4

### Hemodialysis patients’ health-related quality of life

According to the results, the mean KDQOL of CKD patients was 48.9 ± 23.3 (in a possible range of 0 to 100). The mean scores of the domains of KDQOL-SF™ in a range of 0 to 100 are shown in Table 4.

**Table 4:** The mean score of patients quality of life based on kidney disease quality of life short form (KDQOL-SF)

Items	Range	Mean	SD	Minimum score	Maximum score
Symptom/problem list	0–100	74.9	21.2	0	100
Effects of kidney disease	0–100	66.9	21.3	0	100
Burden of kidney disease	0–100	45.1	21.1	0	100
Sum (SF-12)	0–100	40.6	24.8	0	100
Total score on KDQOL-SF	0–100	48.9	23.3	0	100

### Correlation of patients’ perception on benefits and barriers of exercise with their QOL

Data analysis by Pearson correlation coefficient showed a positive and significant relationship between the mean scores of DPEBBS and the total score of KDQOL (*r* = 0.55, *p* < 0.001). Moreover, there was a positive relationship between the mean scores of DPEBBS and the mean score of all domains of KDQOL-SF™ (*p* < 0.00) (Table 5).

**Table 5:** The correlation between mean score of exercise benefits and barriers scale (DPEBBS) and kidney disease quality of life short form (KDQOL-SF)

	DPEBBS^a^ scale	Sum (SF-12)	Burden of kidney disease	Symptom/problem list	Effects of kidney disease	Total score of KDQOL-SF^b^
**DPEBBS**^a^ **scale**	**–**					
**Sum (SF-12)**	***R*** **= 0.42** ***P*** < 0.001^*^	**–**				
**Burden of kidney disease**	***R*** **= 0.33** ***P*** < 0.001^*^	***R*** **= 0.42** ***P*** < 0.001^*^	**–**			
**Symptom/problem list**	***R*** **= 0.41** ***P*** < 0.001^*^	***R*** **= 0.37** ***P*** < 0.001^*^	***R*** **= 0.29** ***P*** < 0.001^*^	**–**		
**Effects of kidney disease**	***R*** **= 0.49** ***P*** < 0.001^*^	***R*** **= 0.45** ***P*** < 0.001^*^	***R*** **= 0.51** ***P*** < 0.001^*^	***R*** **= 0.73** ***P*** < 0.001^*^	**–**	
**Total score of KDQOL-SF**^b^	***R*** **= 0.55** ***P*** < 0.001^*^	***R*** **= 0.81** ***P*** < 0.001^*^	***R*** **= 0.65** ***P*** < 0.001^*^	***R*** **= 0.80** ***P*** < 0.001^*^	***R*** **= 0.82** ***P*** < 0.001^*^	**–**

^a^ Exercise benefits and barriers scale

^b^ kidney disease quality of life short form

^*^ Correlation is significant (*P* < 0.001)

## Discussion

Hemodialysis, as renal replacement therapy, could lead to changes in QoL and the health status of patients with CKD [19]. In this regard, exercise and physical activity could influence the physical, psychological and social factors of patients undergoing hemodialysis [40]. One of the influential factors, which has recently been of interest is the hemodialysis patients’ perception of exercise benefits and barriers [29].

The results of the present study showed that the most common cause of CKD in patients undergoing hemodialysis was hypertension (30%) followed by diabetes (26.8%). This result is consistent with the results of other studies [7, 41, 42].

The findings of the current study showed that the mean score of DPEBBS was 68.2 ± 7.4 (In a range of 24 to 96) and the results indicated that patients have a greater perception of the exercise benefits. In a study in Turkey, Dilek TAŞ et al. [43] showed that the mean DPEBBS score in patients undergoing hemodialysis was 82.9 ± 9.1, which score is greater than the result of our study.

According to the results, the three most important benefits of exercise from the participants’ perception included “exercise improves my mood”, “exercise prevents muscular wasting”, and “exercise improves my appetite”. This finding is in line with a study carried out in Australia by Jayaseelan et al. [13]; the majority of participants (more than 50%) believed that exercise improves their morale, prevents muscle weakness, and improves their appetite.

According to the results of the present study, 74.9% of the samples were able to perform their daily activities independently. However, despite their greater perception of exercise benefits, the majority of them (63.9%) do not engage in any exercise which may be related to perceived barriers of exercise in hemodialysis patients. The most important barriers of exercise identified by DPEBBS included frequent tiredness, frequent lower-extremity muscle fatigue, and fear of arteriovenous fistula injury. These results are similar to the results of a study conducted by Zheng et al. [23] in China. They found factors such as fatigue, physical pain, and fear of fistula injury as main barriers to exercise. In a study by Jayaseelan et al. [13], only 20% of respondents had a concern for arteriovenous fistula as an exercise barrier. Moreover, our finding is inconsistent with a study by Arian et al. [19] aimed to identify barriers to and motivations to exercise in patients undergoing hemodialysis. They reported the worry about getting thirsty during exercise (64%), kidney disease (62%), and fear of falling during exercise (51%) as three main barriers to exercise. In another study, Martins et al. [44] studied hemodialysis patients’ perceptions of exercise benefits and barriers in Brazil. The fear of falling during exercise, patients’ perceptions of the negative impact of exercise on their health, and having kidney disease (92%) were reported as barriers to exercise. Moreover, the majority of participants (90%) regarded fatigue as the main barrier to exercise. In a study in Italy, Hannan and Bronas [45] conducted a study to determine barriers to regular exercise in adult CKD patients with ESRD. They found that fatigue is the most common barrier to exercise. Moreover, fatigue has been perceived as the exercise barrier in other studies [32, 37, 46]. These results support our findings.

According to two open-ended questions of DPEBBS, some participants reported other barriers to exercise such as lack of exercise facilities at home or near their home and lack of encouragement by hemodialysis staff to exercise. In this regard, Tentori et al. [47] showed that offering exercise programs by dialysis facilities increases 38% of the likelihood of engaging patients in the exercise program. Wang et al. [48] conducted a study in the United States and found the lack of exercise equipment (86.2%) and lack of support of the health care team working in hemodialysis centers (93.1%) as barriers to exercise. In a recent study, Clarke et al. [20] suggested that changes in health care professionals’ (HCPs) behavior and over-arching policy could support the engagement and implementation of exercise among patients with ESKD.

In a study in the United States, Kendrick et al. [31] assessed the attitudes, motivations, and barriers to exercise among CKD patients and found the lack of family support and extra burden on the family when doing outdoor exercise as a barrier to exercise; this result is not in line with our results.

The results of the present study also showed that the mean score of KDQOL in CKD patients was 48.9 ± 23.3 (Range: 0 to 100). The highest score is related to the symptoms/problems domain (74.9 ± 21.2) and the lowest score is related to the health status domain (SF-12) (40.6 ± 24.8). Thenmozhi et al. [49] conducted a study in India to assess the QoL of 130 patients undergoing hemodialysis by using the full version of KDQOL-SF™1.3. In their study, the highest score was related to the domain of staff encouragement (84.0 ± 14.8) and the lowest score was related to the domain of burden of kidney disease (38.0 ± 12.8). It seems that these differences can be attributed to the differences in cultural and health care systems.

The analysis revealed a positive and significant relationship between the mean score of DPEBBS with the score of all domains and the total score of KDQOL-SF. This means that the higher perception of exercise benefits leads to a better health-related quality of life. It seems that patients’ perception of exercise benefits could affect their behavior and encourages them to do exercise activities. In this regard, Zamanzadeh et al. [50] studied the effect of physical exercise on QoL of hemodialysis patients and showed that exercise improves QoL of these patients. Moreover, the positive effect of exercise has been reported in other studies [51–53]. In a recent study, Jiménez et al., [54] found that patients on HD with impaired activity levels showed worse HRQoL scores.

Jhamb et al. [21] argue that optimizing exercise participation in patients on hemodialysis could improve the physical well-being and HRQOL of these patients. However, Parson et al. [55] assessed the effect of the exercise program on the effectiveness of dialysis, blood pressure, and QoL of patients with ESRD. The results showed no significant changes in the patients’ QoL. Chu and McAdams-DeMarco [56] argue that a high burden of inactivity and retention of uremic toxins among patients with ESKD impact on cognitive function of patients. Therefore, exercise could play a main role in preventing cognitive decline in these patients. In a qualitative study, Heiwe et al. [57] found that the levels of physical activity in patients with CKD are affected not only by patients’ health status but also, by perceptual factors such as perceived positive and negative well-being status and patients’ self-efficacy.

There are some limitations in this study. We used a verbal report of exercise that is not always accurate. It is recommended to use an objective measure such as pedometers or accelerometers in future studies. Another limitation is the cross-sectional design of this study which precludes the causality of the observed associations. Moreover, in this study, we only included hemodialysis patients. Thus, further studies are recommended to investigate the benefits and barriers of exercise from the perspective of health care professionals and patients’ families. Moreover, conducting qualitative studies could help to provide a deep understanding of the barriers and benefits of exercise among hemodialysis patients. Furthermore, it is recommended to do a similar study on patients of peritoneal dialysis.

## Conclusion

The results of the present study revealed that most patients undergoing hemodialysis had a positive perception of exercise benefits and stated that exercise can be beneficial to their health. Despite their greater perception of the exercise benefits, the majority of them do not engage in any exercise, which indicates that there are barriers to exercise. There were some barriers to exercise such as frequent tiredness, lower-extremity muscle fatigue, and fear of arteriovenous fistula injury which could be considered in providing care for a patient on hemodialysis. The result showed a positive and significant relationship between the mean DPEBBS score with the mean score of each domain and the total score of KDQOL. Participants also referred to factors such as unavailability of exercise equipment at home or near their home. Moreover, the results showed that the staffs working in hemodialysis centers do not encourage patients to engage in exercise. Therefore, it is suggested to improve patients’ exercise attitudes by increasing their awareness of exercise benefits and removing barriers to exercise. Moreover, providing exercise facilities and encouraging the patients by the health care provider to engage in exercises could help the patients to engage in the exercise programs. Additionally, the incorporation of exercise professionals into health care programs of patients on hemodialysis will ensure the quality of physical activities provided by those best-qualified professionals.

## Data Availability

The datasets used and/or analysed during the current study available from the corresponding author on reasonable request.
